# Application of Cyanobacteria *Arthospira platensis* for Bioremediation of Erbium-Contaminated Wastewater

**DOI:** 10.3390/ma15176101

**Published:** 2022-09-02

**Authors:** Nikita Yushin, Inga Zinicovscaia, Liliana Cepoi, Tatiana Chiriac, Ludmila Rudi, Dmitrii Grozdov

**Affiliations:** 1 Department of Nuclear Physics, Joint Institute for Nuclear Research, 141980 Dubna, Russia; 2 Doctoral School Biological, Geonomic, Chemical and Technological Science, State University of Moldova, MD-2028 Chisinau, Moldova; 3 Department of Nuclear Physics, Horia Hulubei National Institute for R&D in Physics and Nuclear Engineering, 077125 Bucharest, Romania; 4 Laboratory of Physical and Quantum Chemistry, Institute of Chemistry, MD-2028 Chisinau, Moldova; 5 Laboratory of Phycobiotechnology, Institute of Microbiology and Biotechnology, MD-2028 Chisinau, Moldova

**Keywords:** erbium, spirulina, bioremediation

## Abstract

Erbium belongs to rare earth elements critical for industry, especially nuclear technology. Cyanobacteria *Arthospira platensis* was used for Er(III) removal from wastewater by applying biosorption and bioaccumulation processes. The influence of pH, Er(III) concentration, contact time and temperature on the biosorption capacity of *Arthospira platensis* was determined. The optimal conditions for Er(III) removal were defined as pH 3.0, time 15 min and temperature 20 °C, when 30 mg/g of Er(III) were removed. The kinetics of the process was better described by the pseudo-first-order model, while equilibrium fitted to the Freundlich model. In bioaccumulation experiments, the uptake capacity of biomass and Er(III) effect on biomass biochemical composition were assessed. It was shown that Er(III) in concentrations 10–30 mg/L did not affect the content of biomass, proteins, carbohydrate and photosynthetic pigments. Its toxicity was expressed by the reduction of the lipids content and growth of the level of malonic dialdehyde. Biomass accumulated 45–78% of Eu(III) present in the cultivation medium. Therefore, *Arthospira platensis* can be considered as a safe and efficient bioremediator of erbium contaminated environment.

## 1. Introduction

Er(III) is a rare earth elements, which is of critical importance for industry. Today erbium compounds are used in metallurgy as additives, in space technology for laser production and in nuclear technology to prepare nuclear reactor control rods [1,2]. Mining, processing and the use of Er(III) compounds led to the generation of large volumes of wastewater, which are released into the environment, resulting in natural water contamination and causing detrimental effects on water organisms [3]. The effect of Er(III) on living organisms is still relatively unexplored. Thus, results presented in [4] showed that the chronic exposure of daphnids to Er(III) reduced their survival, growth and reproduction.

At the same time, wastewater containing Er(III) are important sources of this element, most especially due to its high price, critical importance for industry and limited levels on Earth. Since large volumes of the wastewater containing rare earth elements, including Er(III), are generated, it is important to develop efficient and ecologically safe techniques of their recovery.

A variety of methods have been applied for rare earth elements, including Er(III), recovery, such as chemical precipitation, flotation, electrocoagulation, solvent extraction, ion-exchange and membrane filtration [3,5]. A careful examination of the literature showed that ion-exchange and solvents extraction are the most commonly applied techniques for rare earth elements extraction [2,3,4,6]. However, these techniques are facing some problems, such as the high consumption of solvents and other chemicals, the low selectivity and purity of the extracted elements and low efficiency of metals recovery from very diluted wastewater [5,6]. Furthermore, the risks of secondary environment pollution and possible negative impact on workers’ health have reduced the attractiveness of mentioned processes [7].

To overcome these problems, much attention was directed to microbial technologies, since microorganisms can resist the effect of high concentrations of toxic elements, are able to neutralize their toxic effect, possess high metal uptake capacity and can consequently measurably reduce pollutant concentrations in water [8,9]. The advantage of biological techniques over physical or chemical methods consists of their cost-effectiveness, non-invasiveness and environmental safety [10]. Microorganisms are the unique owners of various metabolic pathways that utilize toxic compounds as a source of energy for cell processes through fermentation, respiration and co-metabolism. They have evolved several mechanisms, such as adsorption, accumulation, the release of extracellular polymeric substances, redox processes for maintaining homeostasis and resistance to heavy metals in order to survive in toxic environments [11]. Bioaccumulation is defined as a metabolism driven functional process in which metal ions are taken into living cells [12]. Bioaccumulation is a complex process which occurs in two stages: the metabolism-independent sorption of metal ions and the intracellular uptake of metal ions [12,13]. In biosorption experiments, living or dead microorganisms can be used. This process is very quick and efficient, since it is a simple operation independent of energy and does not require additional nutrients in case of accumulation [11,12,13,14].

Even microorganisms of different taxonomic groups are successfully applied to degrade metal ions into less toxic forms or eliminate them from the environment [7,8,10,15,16]; however, in the literature there is no information about their application for Er(III) recovery.

For the first time, the bioremediation ability (biosorption and bioaccumulation) of cyanobacteria *Arthospira platensis* (*A. platensis*) toward Er(III) was tested. In biosorption experiments, the effect of pH, Er(III) concentration, time and temperature on biomass sorption capacity was evaluated and the kinetics, thermodynamics and equilibrium of the sorption were described. In bioaccumulation experiments, the accumulation capacity of the biomass as well as the Er(III) effect on the biomass biochemical composition were evaluated.

## 2. Materials and Methods

### 2.1. Materials and Reagents

The chemicals used in experiments (Er_2_(SO_4_)_3_·8H_2_O, NaOH, HNO_3_, Na_2_CO_3_, Na_3_C_6_H_5_O_7_, Folin-Chocolate reagent, Anthracene-9(10H)-one, H_2_SO_4_, phosphovanillin reagent, chloroform, ethanol and thiobarbituric acid) and purchased from Sigma-Aldrich (Darmstadt, Germany) were of analytical grade.

### 2.2. Object of Study

The biosorption and bioaccumulation experiments were carried out with cyanobacteria *Arthospira platensis* CNMN-CB-02 (spirulina) from the collection of non-pathogenic microorganisms (Institute Microbiology and Biotechnology, Chisinau, Moldova). For biomass cultivation the medium with the following composition, major components (in g/L), NaNO_3_—2_._5; NaHCO_3_—8_._0; NaCl—1.0; K_2_SO_4_—1_._0; Na_2_HPO_4_—0.2; MgSO_4_·7H_2_O—0.2; in 1 mL/L of solution of microelements (mg/L): H_3_BO_3_—2.86; MnCl_2_·4H_2_O—1.81; CuSO_4_·5H_2_O—0.08; MoO_3_—0.000015; FeEDTA—1 mL/L, was prepared. Biomass was grown at temperature of 25–28 °C, illumination ~37 µM photons/m^2^/s and pH 8–9 during first two days of growth, and a temperature of 30–32 °C, pH −9–10 and illumination ~55 µM photons/m^2^/s throughout next four days of cultivation [17].

### 2.3. Biomass Characterization

Spirulina biomass used as a biosorbent was characterized using several analytical techniques. Thus, its chemical composition was determined using neutron activation analysis at the IBR-2 reactor (Joint Institute for Nuclear Research, Dubna, Russia). The procedure of biological samples irradiation and analysis can be found elsewhere [18,19,20]. To determine elements with short-lived isotopes (Mg, Al, Cl, Ca, Mn, and I) were irradiated for 3 min at a neutron flux of 1.6·10^12^ n cm^−2^ s^−1^. The induced gamma activities were measured for 15 min by using Canberra HPGe detectors (Mirion Technologies, Atlanta, GA, USA). The samples for determination of elements with long-lived isotopes (Na, Sc, Cr, Fe, Co, Ni, Zn, As, Se, Rb, Br, Sb, Ba, Cs, and U) were irradiated for 4 days at a neutron flux of 3.1·10^11^ n cm^−2^ s^−1^ and induced gamma activities were measured during 30 min and 90 min after 4 and 20 days of decay, respectively. The spectra recording, analysis and concentrations calculations were performed by using Genie 2000 (Mirion Technologies, Atlanta, USA). and software developed in JINR. Functional groups on the spirulina surface were detected by infrared spectrometer on Bruker Alpha Platinum-ATR spectrometer (Bruker Optics, Ettingen, Germany). A total of 24 scans were performed per sample in the range of 4000–400 cm^−1^. The morphology of the spirulina surface was studied using a scanning electron microscope Quanta 3D FEG (FEI, Hillsboro, OR, USA).

### 2.4. Biosorption

For biosorption experiments, biomass cultivated according to Yushin et al. [17] was filtrated, dried and homogenized. Then 100 mg of the biosorbent were added to a 20 mL volume of erbium solution in 50 mL conical flasks. Predetermined values of pH were achieved using nitric acid (0.01 N and 0.1 N) or sodium hydroxide (0.01 N and 0.1 N). The effect of time (3–120 min), Er(III) concentration (10–100 mg/L) and temperature (20–50 °C) on biosorption were investigated. At the end of experiments the biomass was separated from supernatant by filtration, and total erbium concentration in solutions was determined using ICP-OES PlasmaQuant PQ 9000 Elite spectrometer (Analytik Jena, Jena, Germany). The biomass sorption capacity (mg/g) and the efficiency of Er(III) removal (%) were calculated by formulas described elsewhere [17].

(1) $$q=\frac{V\left(C_{i}-C_{f}\right)}{m}$$

(2) $$E=\frac{C_{i}-C_{f}}{C_{i}}\times 100$$

where V is the volume of the solution, ml; C_i_ and C_f_ are the initial and final metal concentrations in mg/L; and m is the mass of sorbent in g.

### 2.5. Bioaccummulation

In order to assess the bioaccumulation capacity of spirulina biomass Er(III) in concentrations of 10–30 mg/L, was added to the cultivation medium on the first day of biomass growth. Then, biomass was cultivated for six days at a temperature of 25–28 °C, illumination ~37 µM photons/m^2^/s and pH 8–9. At the end of the cultivation cycle, the spirulina biomass was separated from the culture liquid by filtration. The amount of biomass, expressed in g/L, was determined spectrophotometrically based on the calibration curve. The samples were standardized to the concentration of 10 mg/mL distilled water. For the biochemical tests, the biomass was subjected to the repeated freezing–thawing procedure. The concentration of Er(III) in experimental solutions was determined by ICP-OES.

As control in both type of experiments served spirulina biomass cultivated in the medium without the addition of Er(III).

### 2.6. Biochemical Tests

Protein content was determined based on the Lowry method and calculated based on the calibration curve plotted for bovine serum albumin [21]. Carbohydrates content was determined using Anthracene-9(10H)-one. The quantitative calculation was performed based on the calibration curve plotted for glucose [18]. The quantitative determination of lipids was performed using the phosphovanillin reagent and calculation was performed based on the calibration curve constructed for oleic acid [22]. The content of phycobiliproteins in the biomass was calculated using formulas based on molar coefficients for pigments. The absorbance of the extract at 620 nm was recorder for phycocyanin and at 650 nm for allophycocyanin [23]. The content of chlorophyll a and β-carotene in the biomass was determined in ethanolic extracts of biomass. Chlorophyll content was determined at the absorbance of 665 nm and β-carotene at an absorbance of 450 nm. The quantitative calculation was performed based on the calibration curves.

The determination of the content of malonic dialdehyde (MDA) in the biomass was carried out based on the reactive substances of thiobarbituric acid (TBA). The content of MDA in samples was calculated using the extinction coefficient of the complex product of the MDA-TBA [24].

### 2.7. Antioxidant Activity

The antioxidant activity of the ethanolic and water extracts was determined using the radical cation ABTS (2,2 azinobis 3-ethylbenzothiazoline-6-sulfonic acid) [25]. The ethanolic extract from the biomass was obtained in a similar way to the extract for the determination of pigments. The water extract was obtained identically to the extracts for the determination of phycobiliproteins. ABTS radical cation was obtained by oxidation with potassium persulfate. ABTS stock solution was prepared by mixing 7 mM ABTS in deionized water with 2.45 mM K_2_S_2_O_8_ at a 1:1 ratio. The ABTS oxidation process took place in the dark at room temperature for 12–16 h. The working radical ABTS solution has an absorbance value of 0.700 ± 0.020 at 734 nm. The samples were obtained by mixing 0.3 mL of biomass extract and 2.7 mL of ABTS solution. The absorbance of the samples was measured after 6 min. The % of inhibition was calculated relative to the absorbance of the ABTS reagent.

### 2.8. Statistical Analysis

The values in the manuscript are presented as average of three experiments ± standard deviation. One-way analysis of variance (ANOVA) was performed using Student’s *t*-test.

## 3. Results and Discussion

### 3.1. Biosorbent Characterization

**Figure 1:**
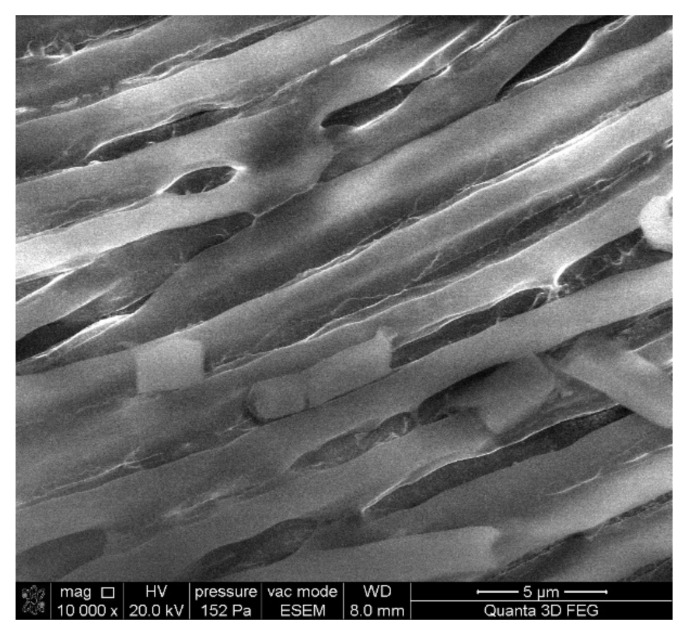
SEM microphotograph of the *A. platensis* biomass.

*A. platensis*, used as a biosorbent, was characterized using several analytical techniques. The scanning electron microscope (SEM) used to visualize the surface morphology of the cyanobacteria biomass (Figure 1) showed straight filaments, which are typical for dead and dry culture. Their length was 20–30 mm and the diameter was 1.5–2.5 mm. From the image it can be seen that most of the filaments are intact, which indicates minimal damage to the biomass during the drying and homogenization process. However, fragmented filaments are also observed but their number is insignificant.

**Figure 2:**
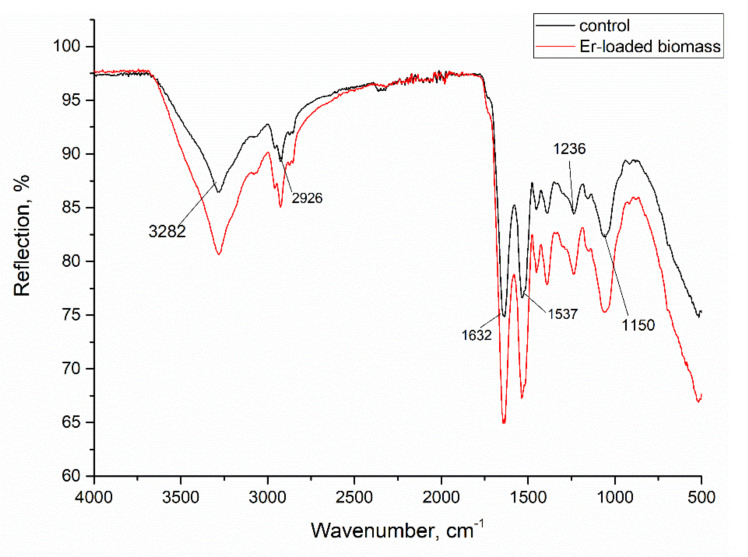
FTIR spectrum of *A. platensis* biomass.

Fourier Transform Infrared Spectroscopy (FTIR) allowed the identification of the functional groups on the spirulina surface, which can participate in metal ion binding (Figure 2). The strong peak at wavenumber 3282 cm^−1^ could be characteristic for hydroxyl and amine functional groups. Stretching vibrations at 2926 cm^−1^ could be attributed to methyl groups, while adsorption peaks in the region 1750–1350 cm^−1^ indicate the presence of –CO groups [26,27]. Signals in the region of 1250–1000 cm^−1^ are characteristic for –C–O, –C–C and –C–OH groups, and in the region 900–500 cm^−1^ they could be attributed to –P–O, –S–O and aromatic –CH groups [28]. In the IR spectra of the Er-loaded biomass, the peaks positions of amine, carboxyl, hydroxyl, phosphate and sulphate groups were shifted by 3–5 cm^−1^, indicating their involvement in Er(III) binding.

Using neutron activation analysis (NAA), the content of 22 elements was determined in *A. platensis* biomass (Table 1). The major elements were Na, K, Ca, Mg, Cl and K, the content of which was on the level of g/kg. NAA allowed the determination of trace elements Fe, Zn, Se, Br, Cr, Ni and I, which play an important role in the metabolism and vital functions of living organisms. Moreover, the content of other elements, such as Al, Sc, As, Rb, Sb, Ba, Cs and U, which have no biological function, was determined. The main source of these elements in the biomass could be considered to be salts used for the preparation of the cultivation medium. It should be mentioned that the content of rare earth elements in the spirulina biomass was below the limit of detection of the NAA technique.

### 3.2. Effect of pH, Time, Temperature and Initial Er(III) Concentration on A. platensis Biosorption Capacity

**Figure 3:**
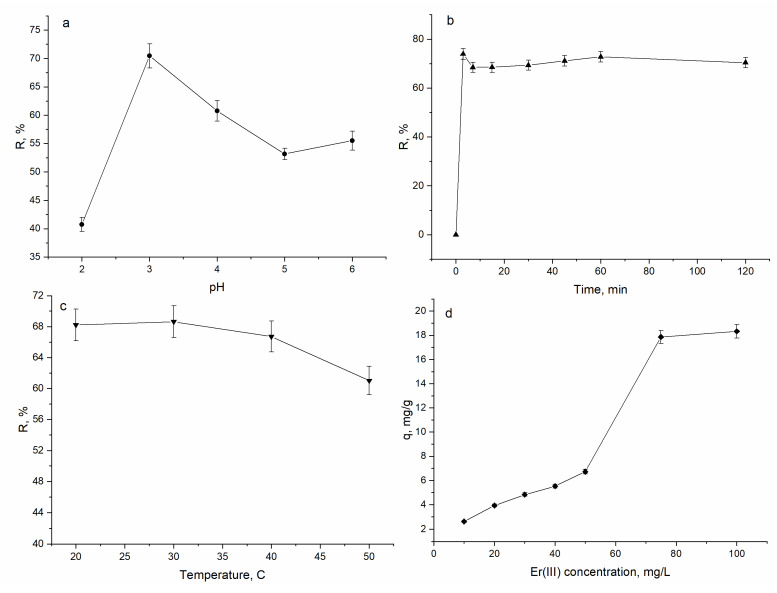
The effect of (**a**) pH, (**b**) time, (**c**) temperature and (**d**) Er(III) concentration on *Arthrospira platensis* biosorption capacity.

The sorption of Er(III) from aqueous solution is dependent of several parameters, among which the most important are the pH of the solution, time of contact, temperature and initial Er(III) concentration in solution. The effect of abovementioned parameters on A. platensis sorption capacity is presented in Figure 3.

The pH of solution is an important parameter in biosorption experiments because of its effect on the chemical forms of metal ions and the charge of the biosorbent surface. The effect of pH on Er(III) removal by A. platensis was studied in the pH range of 2.0–6.0; experiments were not performed at pH values higher than 6.0 due to the formation of Er(OH)_3_. The sorption capacity of spirulina biomass increased from 0.96 mg/g at pH 2.0 to 1.48 mg/g at pH 3.0 and then it was reduced to 1.21 mg/g at pH 6.0. Thus, the maximum removal of Er(III) of 70% took place at pH 3.0. The lowest removal of Er(III) at pH 2.0 (41%) can be explained by a high concentration of H^+^ in solution, which compete with Er(III) for binding sites. With the increase of the pH, the surface of the spirulina biomass become more negatively charged, facilitating metal cations sorption. It should be mentioned that at the pH range of 4.0–6.0 Er(III) removal was on the level of 55–60%. This decrease in Er(III) removal can be explained by the use of NaOH for pH adjusting that resulted in an increase of the concentration of OH groups in solution. Maximum Er(III) removal by activated carbon prepared from rice husk was achieved at pH 4.0 [1] and by zirconia powder at pH 5.0 [29].

The effect of contact time on the removal of Er(III) by A. platensis is presented in Figure 3b. The removal rate was very rapid in the first 3 min of sorbent–sorbate interaction, when 74% of Er(III) was removed from solution. Further increase in contact time did not significantly influence the rate of Er(III) removal and equilibrium was attained. The fast Er(III) biosorption at the initial stage is explained by the availability of abundant active sites on the surface of spirulina, which are filled with time and a plateau is reached [30]. In experiments with activated carbon prepared from rice husks the equilibrium of Er(III) was reached within the first thirty minutes of adsorption [1].

Since the concentrations of pollutants in wastewater may vary greatly, the initial metal ion concentration is a very important factor to be investigated in biosorption studies. The effect of initial Er(III) concentration on biomass biosorption capacity is shown in Figure 3c. An increase in the initial concentration of Er(III) from 10 to 100 mg/L led to the increase of biomass sorption capacity from 1.0 to 12.1 mg/g. Usually it is considered that with the increase of metal concentrations in solution, the number of the available adsorption sites is reduced and this results in a decrease in percentage of metal removal [1]. However, in the present study, the percentage of Er(III) removal at all applied concentration was relatively high, no less than 65%.

Temperature can directly affect the biosorption capacity of spirulina biomass. In the present study, the experiments were performed in the temperature range of 20–50 °C (Figure 3d). It was found that temperature almost did not affect the rate of Er(III) removal. An increase of temperature from 20 to 50 °C resulted in the decrease of spirulina biosorption capacity by 7%, from 68 to 61%. The amount of Er adsorbed on RH activated carbon increased with the solution temperature [1].

### 3.3. Kinetics, Equlibrum and Thermodynamics of the Biosorption Process

In order to assess the changes in biosorption with time two kinetic models: pseudo-first-order and pseudo-second-order kinetic equations were applied.

(3) $$q={\;q}_{e\;}\left(1-e^{-k_{1}t}\right)$$

(4) $$q=\frac{q_{e}^{2}k_{2}t}{1+q_{e}k_{2}t}$$

where q_e_ and q_t_ are the amounts of metal (mg/g) adsorbed at equilibrium and at t (min) time, respectively; k_1_ (1/min) is the rate constant of pseudo-first-order; and k_2_ (g/mg·min) is the rate constant of second order.

The Langmuir and Freundlich isotherm models were used to interpret equilibrium isotherm data.

(5) $$q_{e}=\frac{q_{m\;}{\mathrm{bC}}_{e}}{1+{\mathrm{bC}}_{e}}$$

where C_e_ is metal ions concentration at equilibrium (mg/L), q_e_ is amount of metal adsorbed at equilibrium (mg/g), q_m_ is maximum adsorption capacity of the sorbent (mg/g) and b is Langmuir adsorption constant (L/mg).

(6) $$q_{e}=K_{F}C^{\frac{1}{n}}$$

where K_F_ and n are Freundlich constants, related to biomass sorption capacity.

**Figure 4:**
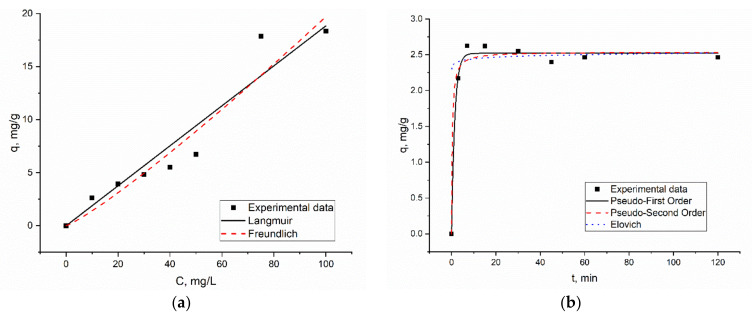
(**a**) equilibrium and (**b**) kinetics of Er(III) biosorption on *A. platensis*.

The graphical presentation of kinetics and the equilibrium of biosorption is presented in Figure 4 and the values of isotherms constants are given in Table 2.

The Freundlich model better represented the data for Er(III) biosorption, the coefficients of determination being higher than for Langmuir model. This tendency may be attributed to the heterogeneous distribution of the sorption sites on the surface of spirulina, since the Freundlich model assumes that sorption occurs on a heterogeneous surface by monolayer adsorption. The applicability of the Freundlich model for the description of metal ions biosorption on A. platensis was shown in other studies as well [31,32]. The maximum sorption capacity calculated from Langmuir model was 30 mg/g and b value indicated the high affinity of Er(III) to spirulina biomass. The adsorption capacity of spirulina was lower than the adsorption capacity of 175 mg/g exhibited by RH-activated carbon [1].

In kinetic studies, good correlation between q experimental and calculated was obtained for both models. Based on their coefficients, the correlation value biosorption of Er(III) on *A. platensis* can be better described using the pseudo-first-order kinetic model. The pseudo-first-order model assumes that the rate of sorption sites filling is proportional to the number of unoccupied sites [30]. The applicability of the pseudo-first-order model for other rare earth elements sorption by spirulina biomass was shown previously [17]. The adsorption of Er(III) onto RH activated carbon was better described by the pseudo-second-order kinetic model [1].

In order to evaluate the thermodynamics of the biosorption process the following parameters, standard free energy, enthalpy and entropy, were estimated.

(7) $$\Delta G^{0}=H^{0}-T\Delta S^{0}$$

(8) $${\mathrm{lnK}}_{d}=\frac{{\Delta S}^{0}}{R}-\frac{{\Delta H}^{0}}{\mathrm{RT}}$$

(9) $$K_{d}=\frac{\left(C_{0}-C_{e}\right)V}{{\mathrm{mC}}_{e}}$$

where ∆G^0^ is free energy (kJ/mol), ∆H^0^ is enthalpy (kJ/mol), ∆S^0^ is entropy (J/mol·K) and K_d_ is the distribution coefficient.

**Figure 5:**
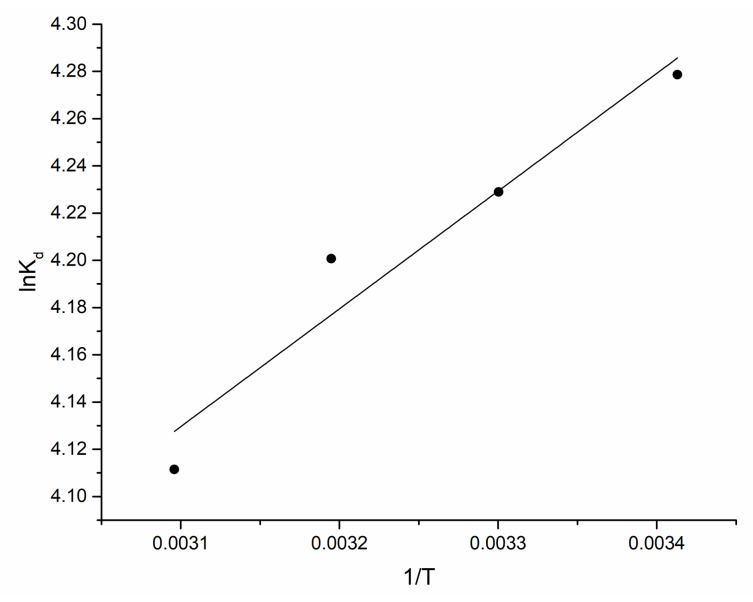
lnK_d_ vs. 1/T.

The values of enthalpy and entropy were calculated from the slope and intercept of the plot of lnK_d_ versus 1/T (Figure 5) and the thermodynamic parameters are presented in Table 3.

Obtained ∆G^0^ values pointed at spontaneous nature of biosorption process. According to literature data, ∆H^0^ in the range of 2.1–20.9 kJ/mol is anindicator of a physical adsorption process, while values of 80–200 kJ/mol are characteristic for chemisorption [30]. The negative charge of ∆H^0^ in the present study indicated an exothermic process, while its values showed that Er(III) biosorption on *A. platensis* was a physical adsorption. The physical nature of the biosorption explains the applicability of the pseudo-first-order model for the description of the kinetics of the sorption. Positive ∆S^0^ values indicated an increase in randomness at the solid/solution interface during adsorption. Since the value of ∆S^0^ was higher than −10 J/mol·K, it can be suggested that the adsorption reaction complies with a dissociative mechanism [33].

### 3.4. Bioaccumulation

The addition of Er(III) in a concentration of 10–30 mg/L resulted in the uptake of 45–78% of ions from solution. The lowest efficiency of uptake was observed at Er(III) of 10 mg/L and it increased with the increase of the metal concentration in the medium.

**Figure 6:**
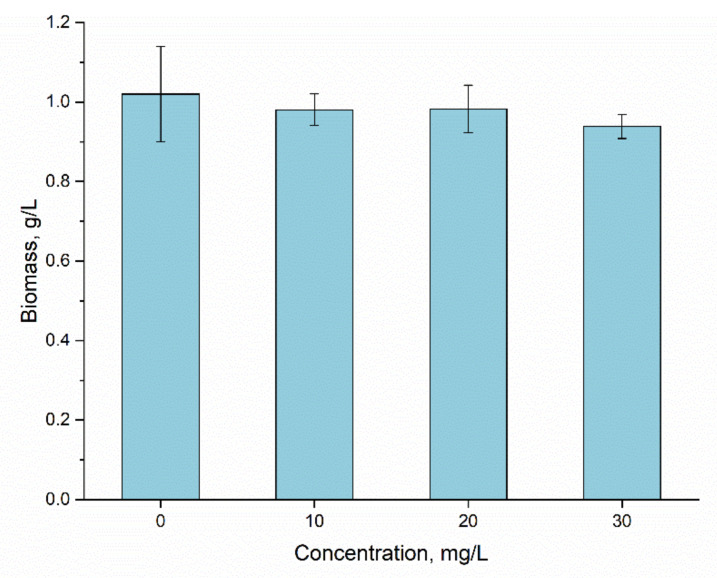
The effect of Er(III) at concentrations 10–30 mg/L on *A. platensis* biomass.

Applied Er(III) concentrations did not affect the accumulation of spirulina biomass (Figure 6). Thus, the amount of biomass accumulated in the control sample was 1.02 ± 0.12 mg/L, and in the experimental variants it was within the limits of 0.94–0.98 g/L dry biomass. The statistical analysis did not reveal any significant differences between control and experimental samples.

Spirulina can react very differently to the presence of rare earth elements in the cultivation medium, and the effect can range from inhibition to neutral and up to a stimulating one [17,34,35,36,37,38]. The effect of Er(III) observed in the present study was very similar to that of Sm, Tb and Nd, which in concentrations of 10–30 mg/L did not affect the amount of spirulina biomass. Likewise, no significant changes in the amount of biomass were observed when the effect of Eu on spirulina in the same range of concentrations was studied [17,34].

It was demonstrated that the toxic effect of some xenobiotics on cyanobacteria, including *A. platensis*, can be manifested even though there is no statistically significant decrease in the amount of biomass [17,34]. In this case, it is important to monitor the biochemical composition of the spirulina biomass cultivated on the medium to which the respective xenobiotic was added—in our case, erbium.

**Figure 7:**
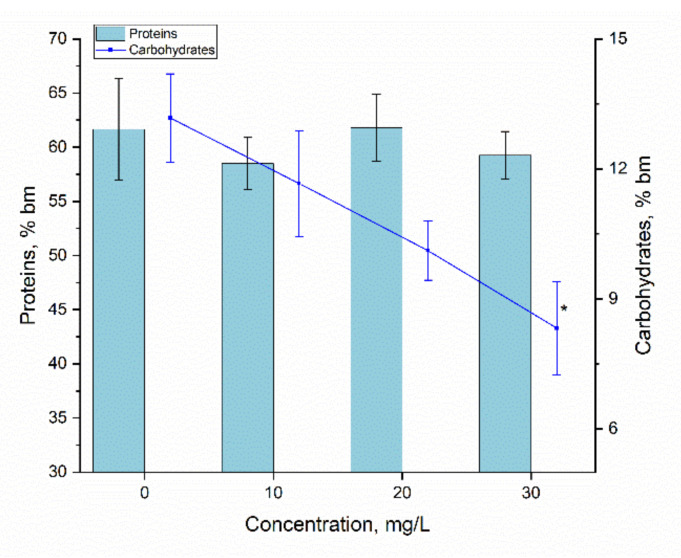
The content of proteins and carbohydrates in *Arthrospira platensis* exposed to Er(III) at concentrations 10–30 mg/L (* − *p* = 0.039 for the difference between control and experimental sample—carbohydrates).

Since *Arthrospira platensis* is a valuable source of proteins, their content in biomass is a key indicator of both the quality of the biomass and the level of biomass impairment under different stress factors. The results obtained for this indicator can be seen in Figure 7.

The statistical analysis did not reveal significant difference in protein content in spirulina biomass grown in the presence of 10–30 mg/L of Er(III), the *p* values being in the range of 0.095–0.46, which is below the acceptable threshold of 0.05. Thus, from the point of view of protein content, spirulina responded neutrally to the presence of Er(III) in the medium of growth.

The content of carbohydrates in the spirulina biomass at Er(III) concentrations of 10 and 20 mg/L did not differ from that characteristic for control, and at a concentration of 30 mg/L it was reduced by 36.7% compared to control (*p* = 0.0386). A similar response of the spirulina culture was observed in the case of Eu [17]. The presence of Nd in the cultivation medium also had a negative effect on carbohydrate content in spirulina biomass. It should be noted that this effect was observed at all studied Nd concentrations [34].

**Figure 8:**
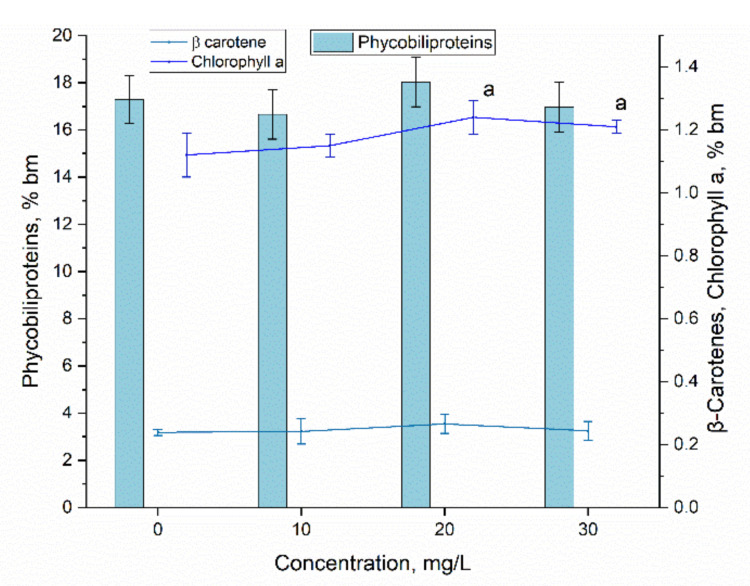
The content of pigments in *Arthrospira platensis* exposed to Er(III) at concentrations 10–30 mg/L, a—*p* < 0.05 for difference between control and experimental sample.

The changes in the content of photosynthetic pigments in spirulina biomass under the influence of Er(III) can be seen in Figure 8. In the control biomass, the content of α-chlorophyll constituted 1.12%; of β-carotene, 0.24%; and that of phycobiliproteins constituted 17.30% of the dry biomass. From the data presented in Figure 8, it can be seen that no severe alterations in the content of photosynthetic pigments took place.

The phycobiliproteins are components of spirulina biomass that react very quickly to the action of compounds with a toxic effect or stressful conditions. For example, in the conditions of saline stress or stress caused by the presence of heavy metals, the amount of phycobiliproteins in biomass can decrease significantly, more than by 10 times, compared to the control [18,20]. The significant decrease (by 6.7–64.5%) of phycobiliprotein content was observed when spirulina was cultivated in the presence of dysprosium, samarium, terbium, lanthanum, neodymium and ytterbium [34]. A situation similar to that observed for Er(III) was noted at addition of Eu(III) in cultivation medium. The applied Eu(III) concentrations did not change the content of phycobiliproteins in the biomass [17].

The content of β-carotene also was not significantly affected by the presence of Er(III) in the cultivation medium. Our previous results obtained for other rare earths (Sm, La, Dy, Nd, Yb) demonstrated that in most cases the content of β-carotene in metal-loaded biomass was on the level of control or increased by up to 25% compared to the control [34]. Only in the case of Eu(III) at concentrations of 30 mg/L did a slight decrease in the content of β-carotene take place [17].

In previous research conducted for other rare earths, the content of chlorophyll α in spirulina biomass was a rather stable parameter. Thus, only Sm (at concentrations 10–30 mg/L) and Eu (at concentration of 30 mg/L) provoked a significant decrease in this pigment content in the biomass [17,34]. In the case of Er(III), spirulina reacted differently, which was manifested by an increase of the content of chlorophyll α by 8.3 and 10.7% (*p* = 0.042 and 0.43, respectively).

The maintenance of the content of photosynthetic pigments at a level characteristic for spirulina grown in the medium without addition of Er(III) confirms biomass adaptation to the presence of erbium in the nutrient medium in the mentioned concentrations. Due to this fact, the experimental biomass productivity was comparable with control values.

**Figure 9:**
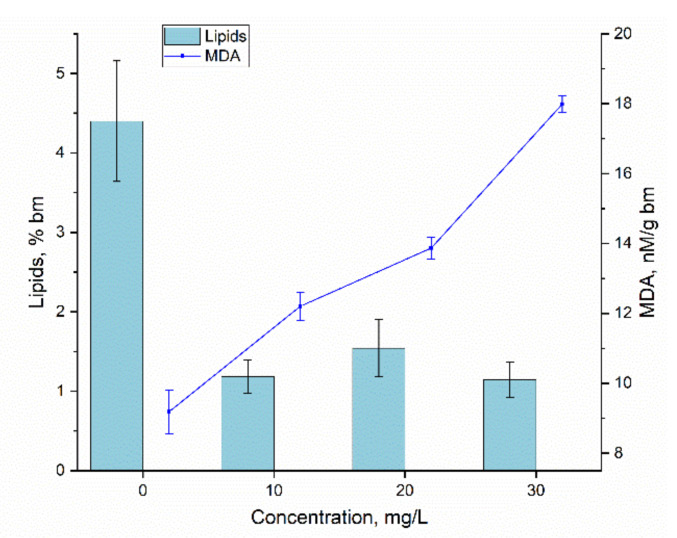
The content of lipids and MDA in *Arthrospira platensis* exposed to Er(III) at concentrations of 10–30 mg/L a—*p* < 0.05 for the difference between control and experimental sample.

However, there were indications of Er(III) toxicity to the spirulina culture. It concerns the content of lipids and malonic dialdehyde, the product of the oxidative degradation of lipids, in the biomass. The results obtained for these two parameters can be seen in Figure 9.

The content of lipids in the control biomass was 4.405 ± 0.76, while in the experimental variants it was slightly more than one percent. Thus, the content of lipids in the dry spirulina biomass decreased 2.85–3.83 times compared to the control. The amount of MDA in the control biomass was 9.18 nM per gram of dry biomass, while in experimental variants the content of this marker of oxidative stress was 1.3–2.0 times higher. These two factors analyzed as a whole are eloquent evidence of a state of oxidative stress created by the presence of Er(III) in the cultivation medium. However, the state of stress was successfully managed by the spirulina cells, which was confirmed by the maintaining of other biochemical parameters within normal limits.

The results on the effect of other rare earth elements on lipids content in spirulina biomass showed a variety of responses—from reactions similar to those caused by Er(III) to diametrically opposite reactions. Thus, for example, La caused a slight increase in the content of lipids, while Tb, Dy and Yb reduced it, whereas Sm and Nd at concentrations of 10–30 mg/L did not influence the total lipid content [34]. Eu(III) like Er(III) led to a significant reduction of total lipid content in spirulina biomass at all studied concentrations [17].

The content of MDA under the action of rare earth elements, except Tb, increased at all given concentrations. A pattern similar to that observed for Er(III) was spotted for Nd, Yb and Eu, when all applied metal concentrations caused a significant increase in MDA content [17,34].

**Figure 10:**
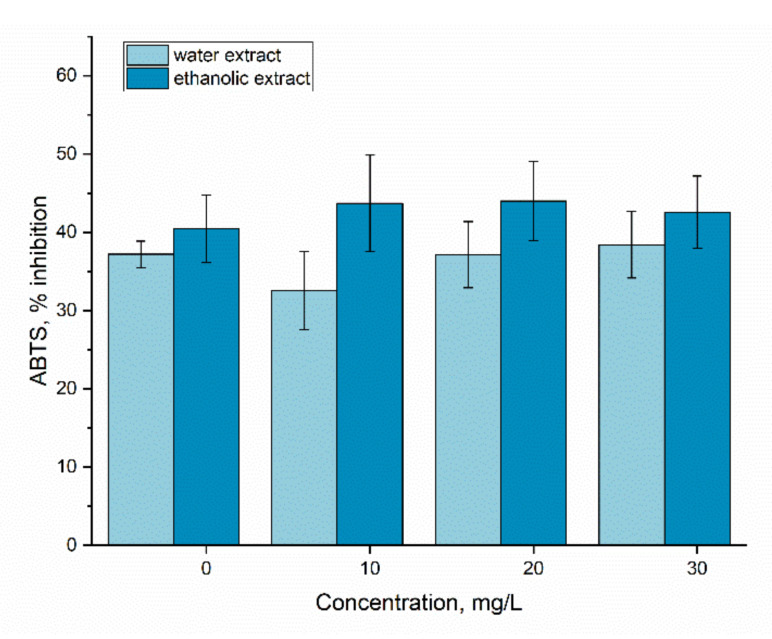
The antioxidant activity of extracts from *A. platensis* biomass exposed to Er(III) at concentrations 10–30 mg/L.

Additionally, the antioxidant activity of the biomass was maintained at a stable level, Figure 10.

The activity of the ethanolic and water extracts from spirulina biomass grown in Er-loaded medium was at the level of the control at all applied Er(III) concentrations. To our knowledge is the first rare earth element which did not cause a change in the inhibition power of the ABTS cation radical. At the addition of Eu in cultivation medium, this parameter increased by more than 30% at the Eu(III) concentration of 10 mg/L and by more than 20% in the case of the concentration of 10 mg/L [17]. The previously obtained data for La, Dy, Sm and Tb also showed a pronounced increase in the antioxidant activity of the aqueous extract of spirulina biomass, while Nd and Yb in the concentration range of 10–30 mg/L caused a considerable decrease of this parameter [34]. In the present study, the maintenance of antioxidant activity at a level characteristic for control biomass confirms spirulina adaptation to Er(III).

## 4. Conclusions

The biosorption of Er(III) on *A. platensis* was dependent on several parameters and the maximum biomass sorption capacity of 30 mg/g was achieved at pH 3.0, temperature 20 °C and 3 min contact time. Freundlich isotherm showed its applicability for the description of equilibrium data, while the kinetics of the sorption was better presented by the pseudo-first-order model. From a thermodynamic point of view, the biosorption process was spontaneous and exothermic (∆H^0^ −4.1 kJ/mol).

In bioaccumulation experiments, 45–78% of Er(III) were removed from solution; moreover, biomass productivity was almost on the level of control, 0.94–0.98 g/L. The level of proteins, carbohydrates (except Er(III) at concentration of 30 mg/L) and pigments was almost not affected by the presence of Er(III) in the cultivation medium. The toxic effect produced by Er(III) was confirmed by a reduction of the level of lipids, 2.85–3.83 times compared to control and the twofold increase of malonic dialdehyde content. The change of these parameters indicate a state of oxidative stress expressed through the oxidative degradation of lipids. Through long-term influence, or on the application of higher concentrations, the toxic effects of Er(III) could become more pronounced and could affect both the productivity and the quality of the spirulina biomass.

## Figures and Tables

**Table 1:** Elemental composition of *A. platensis* biomass determined using neutron activation analysis.

Element	Content, mg/kg	Element	Content, mg/kg
Na	10,600 ± 420	Co	0.12 ± 0.01
Mg	5380 ± 270	Zn	34 ± 2.7
Al	130 ± 6	As	0.44 ± 0.02
Cl	6430 ± 385	Se	0.12 ± 0.03
K	18,600 ± 1670	Br	1.9 ± 0.2
Ca	21,100 ± 2300	Rb	0.32 ± 0.07
Sc	0.01 ± 0.002	Sb	0.06 ± 0.003
Cr	8.9 ± 0.9	I	4 ± 0.7
Mn	117 ± 4.5	Ba	25 ± 1.3
Fe	4610 ± 230	Cs	0.009 ± 0.002
Ni	4.4 ± 0.4	U	0.04 ± 0.003

**Table 2:** Constants of the kinetics and isotherm of Er(III) sorption on *A. platensis*.

	Isotherms					
Model	Langmuir			Freundlich		
Parameters	q_m_	b	R^2^	K_F_	*n*	R^2^
Er(III)	30	0.006	0.90	0.1	0.87	0.98
	Kinetics					
Model	Pseudo-first order			Pseudo-second order		
Parameters	q_e_	k_1_	R^2^	q_e_	k_2_	R^2^
Er(III)	2.5	0.67	0.99	2.5	1.18	0.98

**Table 3:** Thermodynamics parameters of Er(III) biosorption on *A. platensis*.

Temperature, K	∆G^0^, kJ/mol	∆H^0^, kJ/mol	∆S^0^, J/mol·K
293	−10.4	−4.1	21.5
303	−10.6		
313	−10.9		
323	−11.1		

## Data Availability

Not applicable.
